# Molecular characterization of clinical and environmental *Vibrio parahaemolyticus* isolates in Huzhou, China

**DOI:** 10.1371/journal.pone.0240143

**Published:** 2020-10-02

**Authors:** Wei Yan, Lei Ji, Deshun Xu, Liping Chen, Xiaofang Wu

**Affiliations:** Huzhou Center for Disease Control and Prevention, Huzhou, Zhejiang, China; Cornell University, UNITED STATES

## Abstract

*Vibrio parahaemolyticus* is responsible for seafood-borne gastroenteritis worldwide. Isolates of *V*. *parahaemolyticus* from clinical samples (n = 54) and environmental samples (n = 38) in Huzhou were analyzed by serological typing, virulence gene detection, antibiotic resistance testing, and pulsed-field gel electrophoresis (PFGE) for molecular typing. O3:K6 was the main serotype and tlh+tdh+trh- was the most frequently detected virulence genotype in clinical strains. O2:Kut was the main serotype and tlh+tdh-trh- was the most frequently detected virulence genotype in environmental strains. Antibiotic resistance testing indicated that the isolates were highly resistant to ampicillin (90.76%), followed by gentamicin and tetracycline. Following the restriction enzyme *Not*I digestion, the 91 strains yielded 81 PFGE patterns, and 16 clones had similarity values of > 85.00%, indicating a high level of diversity. Finally, there may be cross-contamination between freshwater and seawater products, so it is necessary to strengthen supervision of food processing.

## Introduction

*Vibrio parahaemolyticus* is a Gram-negative haemolyticus widely found in seawater, seafloor sediments, and aquatic products such as fish, shrimp, and shellfish near the coast. It is one of the main pathogens causing infectious diarrhea in coastal areas [1–3] and can cause acute gastroenteritis and primary sepsis. In 1950, *V*. *parahaemolyticus* was discovered in a food poisoning incident in Japan, in which at least 272 people were sickened and 20 died due to ingestion of small sardines that were partially dried [4]. In recent years, rising demand has led more seafood to be transported to inland areas, contributing to an increase in V. parahaemolyticus cases each year [5].

Huzhou is located in inland China, and many residents eat raw or undercooked aquatic products (Fish *et al*.). Therefore, *V*. *parahaemolyticus* has become the most important pathogen causing infectious diarrhea in Huzhou [6]. Despite the high risk of *V*. *parahaemolyticus*, information on its prevalence or molecular epidemiology is not readily available.

We investigated *V*. *parahaemolyticus* strains collected from clinical samples and environmental samples in Huzhou in terms of their serotypes, virulence genes, genotypic traits, antimicrobial resistance, and molecular characteristics. The findings will provide vital information to government agencies for performing risk assessments of *V*. *parahaemolyticus* infection.

## Materials and methods

### Bacterial isolates

A total of 92 strains of *V*. *parahaemolyticus* was isolated from clinical (stool samples) and environmental (water surface samples) collected at food markets during foodborne disease surveillance in 2019 from several regions of Huzhou. The 92 strains of *V*. *parahaemolyticus* included 54 isolates from stool samples and 38 from environmental samples. The 92 isolates were stored at −80°C in trypticase soy broth (TSB) containing 20% glycerol. The quality control strain for pulsed-field gel electrophoresis (PFGE)—Brendan Lupussalmonellae (H9812) [7]—was from the Zhejiang Center for Disease Control and Prevention, China.

Human ethics approval was not requested because no human subjects were involved.

### Serotyping

Serological analysis of the lipopolysaccharide (O) and capsular (K) antigens of the *V*. *parahaemolyticus* isolates was performed by agglutination tests using a commercial *V*. *parahaemolyticus* Antiserum Test Kit (Denka Seiken, Tokyo, Japan). Pure cultures of bacterial strains on 3% NaCl TSA plate were subjected to glass-slide agglutination to detect K antigen. Freshly cultured colonies were ground into sterilized 3% NaCl to produce a bacterial suspension, which was inactivated at 121°C for 1 h and centrifuged at 4000 r/min for 10 min. The supernatant was discarded, and the bacterial suspension was precipitated and resuspended in sterilized 3% NaCl. The bacterial suspension was subjected to O-antigen analysis; normal saline was used as the control.

### Analysis of virulence genes

DNA was extracted by boiling. A ring of fresh colony was ground in 200 μL of sterilized water. The bacterial suspension was bathed at 100°C for 10 min and centrifuged at 10000 r/min for 5 min. The supernatant contained strain DNA was collected and stored at −80°C until use.

Virulence genes of the *V*. *parahaemolyticus* isolates were analyzed by fluorescence PCR using a commercial *V*. *parahaemolyticus* Triple Nucleic Acid Testing Kit (Biogen Biological Co., Ltd., Shenzhen, China), in accordance with the manufacturer’s instructions.

### Antimicrobial susceptibility testing

The antimicrobial susceptibility of the 92 *V*. *parahaemolyticus* isolates was tested using the broth microdilution method. The 10 antibacterial agents were ampicillin (AMP), tetracycline (TET), chloramphenicol (CHL), cotrimoxazole (SXT), ceftazine (CAZ), gentamicin (GEN), imipenem (IMI), ciprofloxacin (CIP), amikacin (AMI), and levofloxacin (LEV). Antimicrobial susceptibility was interpreted as sensitive, intermediate, or resistant, with reference to the Interpretation Criteria for Other non-Enterobacteriaceae in the Clinical and Laboratory Standards Institute (CLSI) Drug Sensitivity Test Guidelines. *Escherichia coli* ATCC 25922 was used as a control. The results were analyzed using the CLSI breakpoints.

### PFGE

PFGE analysis of the *V*. *parahaemolyticus* isolates was performed according to the PFGE Standard Operating Method of *Vibrio parahaemolyticus* [8]. First, chromosomal DNA was digested using the restriction enzyme *Not*I (TaKaRa, Japan). The digestion fragments were subjected to PFGE in a 1% Seakem Gold Agarose gel (Lonza Company, Swiss) in 0.5% Tris-boric acid-ethylenediaminetetraacetic acid (EDTA) buffer using a CHEF Mapper XA system (Bio-Rad Laboratories, Richmond, CA, USA). The electrophoresis conditions were as follows: fragment size 78 to 396 kb, electrophoresis time 19 h, and pulse time 10 to 35 s. The *Xba*I-digested DNA from *Salmonella enterica* serotype Braenderup strain H9812 was used as a standard. The PFGE patterns were analyzed using Chinese Pathogen Identification Net. Clustering was performed using the unweighted pair group method and the Dice correlation coefficient with a position tolerance of 1.5%. Clusters were defined on the basis of a 90% similarity cutoff [9].

### Statistical analysis

Statistical analysis was performed using Microsoft Office Excel 2010 and data are expressed as numbers or percentages of cases.

## Results

### Serotyping

Serological analysis of the 54 *V*. *parahaemolyticus* isolates from clinical samples revealed six serovars with O3:K6 (35 strains, 64.81%), followed in order of frequency by O4:Kut (10 strains, 18.52%). Serological analysis of the 38 *V*. *parahaemolyticus* isolates from environmental samples revealed nine serovars with O2:Kut (eight strains, 21.05%), followed in order of frequency by O5:Kut (seven strains, 18.42%) (Table 1).

**Table 1:** Serotypes of 92 *V*. *parahaemolyticus* isolates from clinical and environmental samples in Huzhou.

	clinical strains		environmental strains	
Serotyping	Number of isolate	The percentage (%)	Number of isolate	The percentage (%)
O3:K6	35	64.82	0	0
O4:K8	1	1.85	0	0
O4:Kut	10	18.52	4	10.53
O3:Kut	6	11.11	4	10.53
O1:Kut	1	1.85	6	15.79
O2:Kut	0	0	8	21.05
O2:K3	0	0	1	2.63
O2:K28	0	0	4	10.53
O5:Kut	1	1.85	7	18.42
O5:K17	0	0	1	2.63
O10:Kut	0	0	3	7.89
Total	54	100	38	100

### Analysis of virulence genes

All of the 92 isolates were positive for *tlh*. Among 54 isolates from clinical samples, 50 isolates had tdh and only 1 isolate had trh. Six isolates from environmental samples had *tdh*, and all of the 38 isolates from environmental samples were negative for *trh* (Table 2).

**Table 2:** Virulence genes of 92 *V*. *parahaemolyticus* isolates from clinical and environmental samples in Huzhou.

virulence genes	clinical strains		environmental strains	
	Number of isolate	The percentage (%)	Number of isolate	The percentage (%)
tlh^+^tdh^+^trh^-^	50	95.80	6	15.79
tlh^+^tdh^-^trh^-^	3	3.36	32	84.21
tlh^+^tdh^-^trh^+^	1	0.84	0	0
Total	54	100	38	100

### Antimicrobial resistance

The majority of the *V*. *parahaemolyticus* strains was resistant to ampicillin (90.76%), compared to 4.35% and 2.17% for gentamicin and tetracycline, respectively. All of the isolates were sensitive to the other seven antibiotics. Additionally, 100% of the isolates were sensitive to chloramphenicol, ceftazine, ciprofloxacin, and levofloxacin (Table 3).

**Table 3:** Antimicrobial susceptibilities of the 92 *V*. *parahaemolyticus* isolates.

Antimicrobial	sensitive (%)	intermediate (%)	resistant (%)
AMP	1.09	8.70	90.21
TET	93.48	4.35	2.17
CHL	100	0	0
SXT	98.91	1.09	0
CAZ	100	0	0
GEN	89.13	6.52	4.35
IMI	100	0	0
CIP	100	0	0
AMI	97.83	2.17	0
LEV	100	0	0

### PFGE and cluster analysis

**Fig 1:**
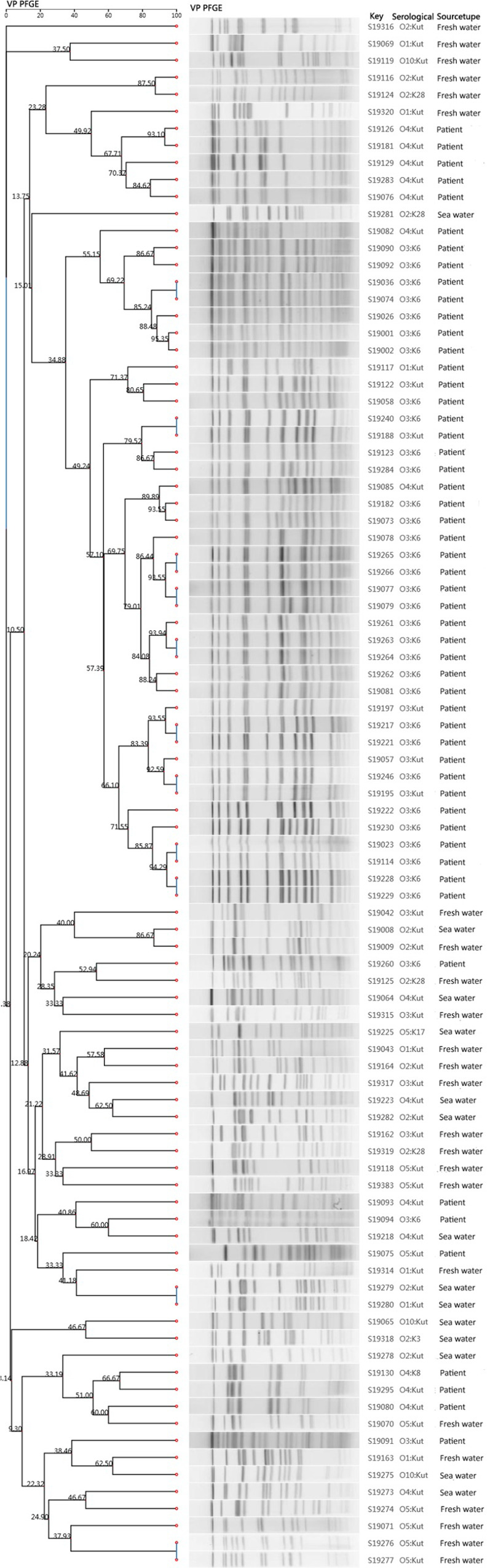
PFGE of *Not*I-digested genomic DNA of selected clinical and environmental *V*. *parahaemolyticus* isolates. Strain identification number, seromarkers, and sample source.

Genotyping of the 92 *V*. *parahaemolyticus* isolates showed that one was non-typable due to DNA degradation during endonuclease digestion. PFGE analysis of the remaining 91(53 from clinical samples and 38 from environmental samples) isolates yielded 81 distinguishable patterns. Among them, 45 isolates were clustered in 16 patterns with a more than 85% similarity threshold, which contained isolates belonging to serotypes O3:K6 and O4:Kut. The similarity threshold for the other 46 isolates was less than 85.00%. The cluster map showed that the similarity of clinical isolates was higher than that of environmental isolates. Also, the maximum similarity between the clinical and environmental strains was 60.00% (Fig 1).

## Discussion

*V*. *parahaemolyticus* is an important pathogen causing food poisoning and infectious diarrhea. Food poisoning caused by *V*. *parahaemolyticus* has surpassed *Salmonella* and ranks first among bacterial causes of food poisoning [10–12]. The incidence of food-borne diseases caused by *V*. *parahaemolyticus* is related to local seafood farming [13]. Huzhou is known as the land of fish and rice and has a well-developed aquaculture industry. The consumption of aquatic products is increasing, and the food safety issue caused by *V*. *parahaemolyticus* is increasingly important.

Thermolabile hemolysin (TLH) is an important virulence factor of *V*. *parahaemolyticus* [14], together with heat-resistant direct hemolysin (TDH) and heat-resistant related hemolysin (TRH). Clinical, environmental, and food isolates carry the TLH gene, which is species-specific but not a virulence factor [15]. TDH mediates the Kanagawa phenomenon, a determinant of the virulence of *V*. *parahaemolyticus*. TRH and TDH are potentially lethal enterotoxins. In this study, all 92 *V*. *parahaemolyticus* isolates had *tlh*, which was consistent with their biochemical characteristics. Of the clinical isolates, 95.80% were TDH+ and TRH−, suggesting strong pathogenicity, while 84.21% of the environmental isolates were TDH− and TRH−, suggesting relatively weak virulence, as reported by Li Xue [16]. Although some strains do not harbor *tdh* and *trh*, they can cause severe diarrhea [17], so it is necessary to strengthen the monitoring of *V*. *parahaemolyticus* virulence genes.

*V*. *parahaemolyticus* can be classified into 13 thermally stable O-antigen groups (O1–O13) and 71 thermally unstable K-antigen groups (K1–K71). In addition, some strains cannot be serologically classified, among which O3 predominates at present [18]. Here, 92 *V*. *parahaemolyticus* isolates from clinical and environmental samples were classified into 11 serotypes. The serotypes of clinical and food isolates were inconsistent. The serotypes of clinical isolates were principally O3:K6 and O4:Kut. The serotypes of environmental isolates were diverse but mainly O2, similar to the report by Xiuying [19].

The problem of bacterial resistance has become more serious with the widespread use of antibiotics. *V*. *parahaemolyticus* was sensitive to most of the tested drugs, but the resistance rate to AMP was 90.76%, and some strains were resistant to tetracycline and gentamicin. These results are different from those in other regions [20–22], possibly due to use of different antibiotics in different regions. Monitoring drug resistance in *V*. *parahaemolyticus* can guide clinical decision making. As the aquaculture industry expands in Huzhou, the increasing use of antibiotics may promote drug resistance in *V*. *parahaemolyticus*. Therefore, monitoring *V*. *parahaemolyticus* drug resistance is critical.

PFGE is the gold standard for bacterial typing and can analyze the relationship between strains at the molecular level and trace their origins. We found polymorphisms, low genetic similarity, and no obvious epidemiological relationship between the clinical and environmental strains. There were 16 clones with a similarity of more than 85.00%, among which 12 were from clinical samples and 4 from environmental samples. The cluster map showed that the similarity of clinical strains was greater than that of environmental strains. Also, the maximum similarity between the clinical and environmental strains was 60.00%, such as S19094, S19218, S19080, and S19070, suggesting no genotypic homology between the environmental and clinical strains. Notably, the similarity between S19008 (seawater sample) and S19009 (freshwater sample) was 86.67%. The samples were taken from non-adjacent stalls in the same farmers’ market, suggesting the possibility of cross-contamination between freshwater and seawater during transportation or sale. Therefore, the appropriate departments need to strengthen supervision, provide early warning, and reduce the incidence of foodborne diseases caused by *V*. *parahaemolyticus*.

## Conclusions

We analyzed *V*. *parahaemolyticus* isolates from clinical and environmental samples in Huzhou. A variety of serotypes was detected. Although the *V*. *parahaemolyticus* strains were sensitive to most common antimicrobial agents, attention should be paid to the potential emergence of resistant strains and the management of antimicrobials needs to be strengthened. In addition, there may be cross-contamination between freshwater and seawater products, so it is necessary to strengthen supervision of food processing. This information will be useful for the control and treatment of foodborne illnesses caused by *V*. *parahaemolyticus* in Huzhou.

## Supporting information

S1 Raw images (PDF)
