# Supplementary figures and images for: Molecular characterization of clinical and environmental Vibrio parahaemolyticus isolates in Huzhou, China
Source: PLoS One. 2020 Oct 2;15(10):e0240143. doi: 10.1371/journal.pone.0240143 (PMC7531842; doi:10.1371/journal.pone.0240143)

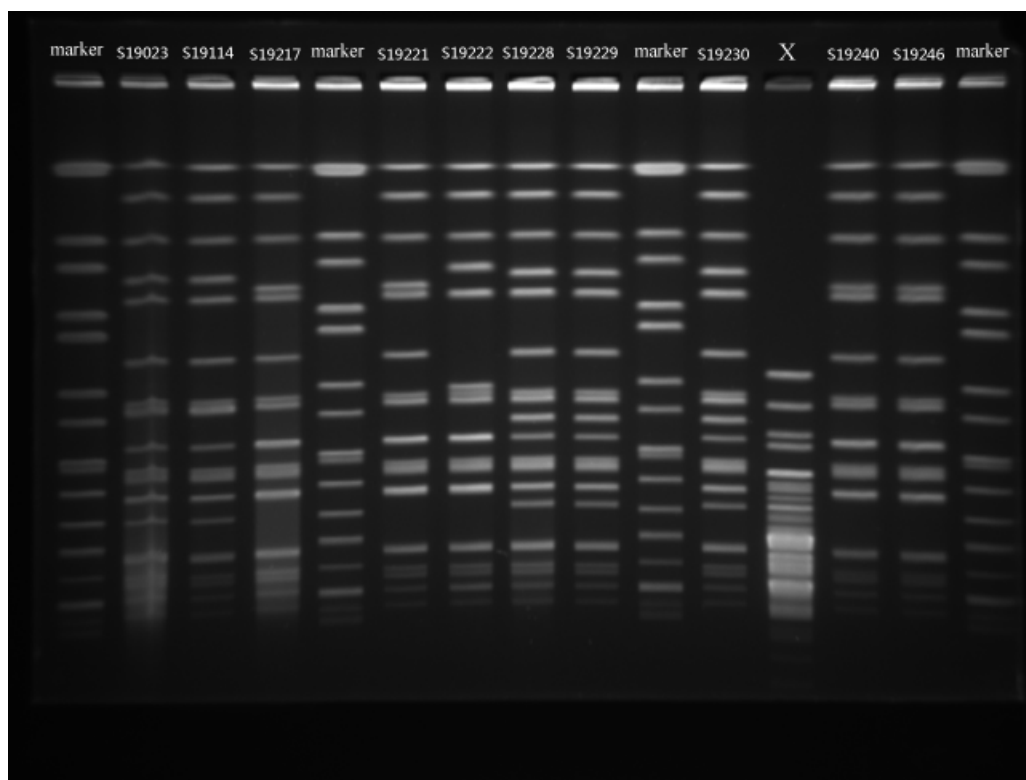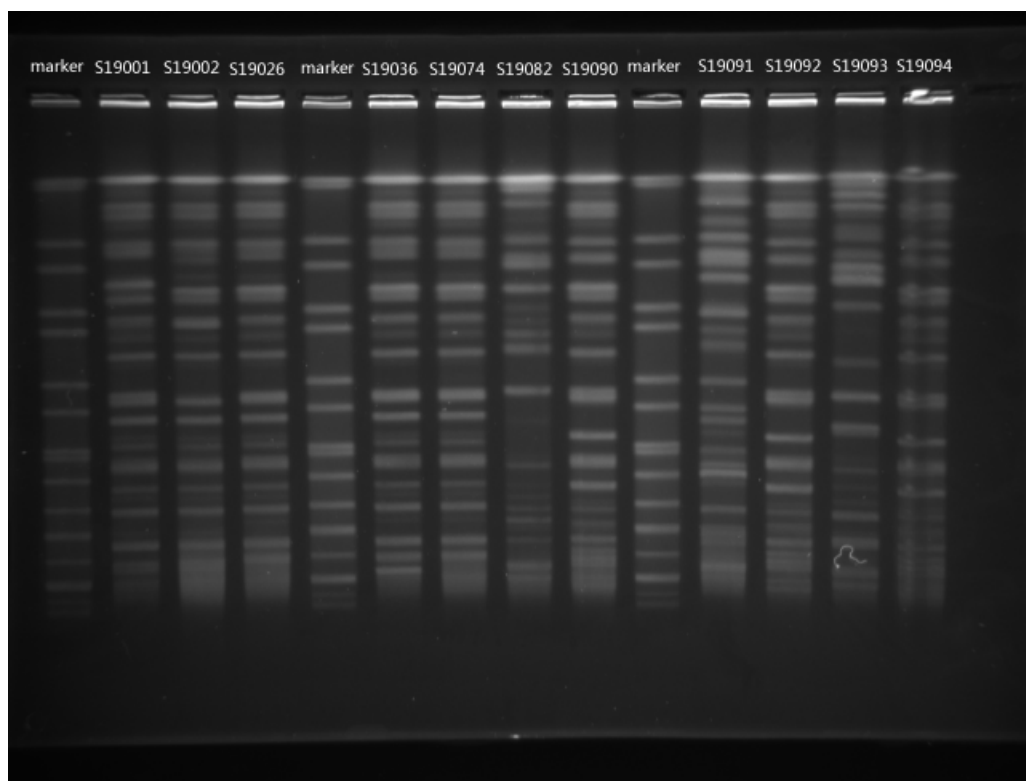

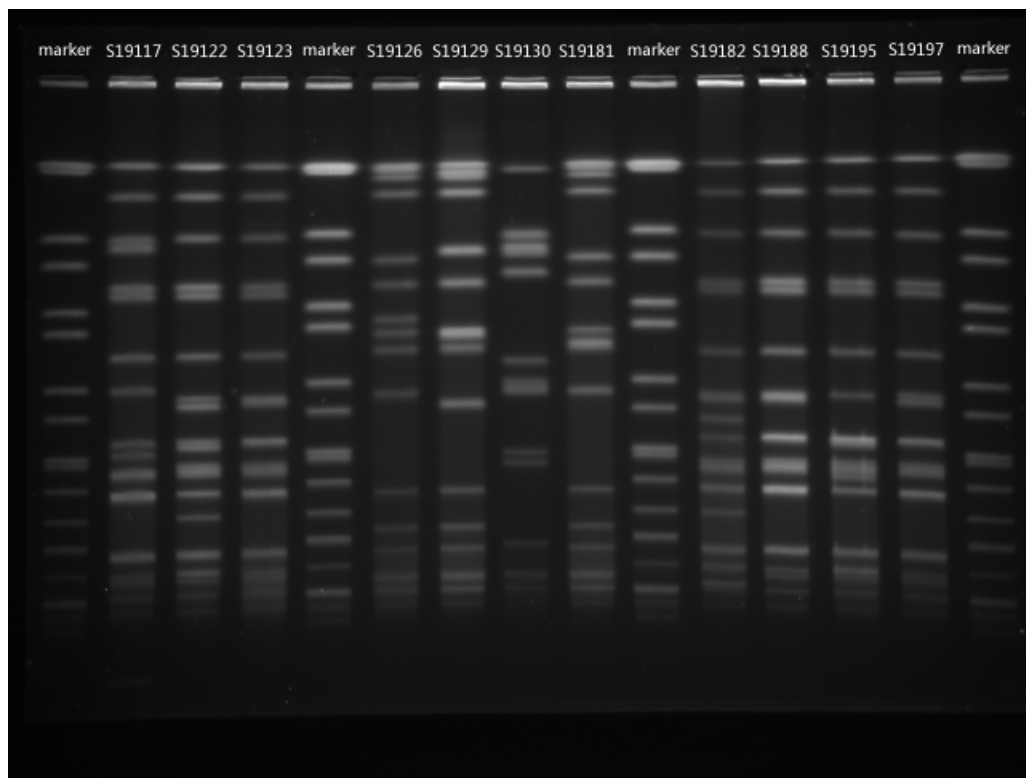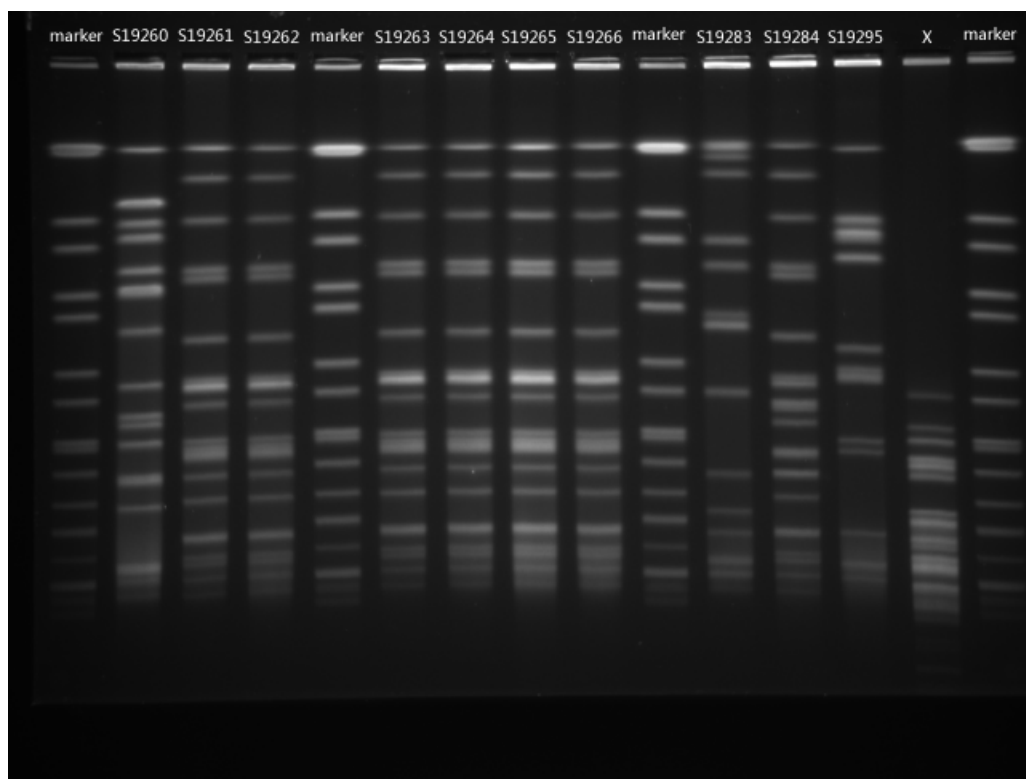

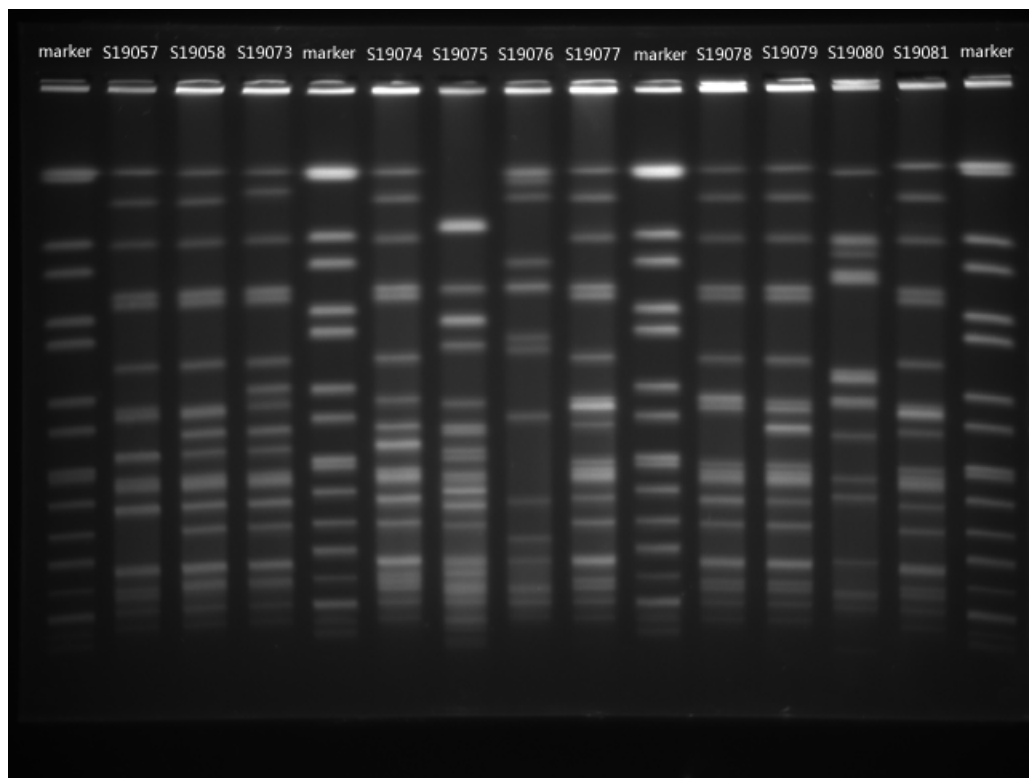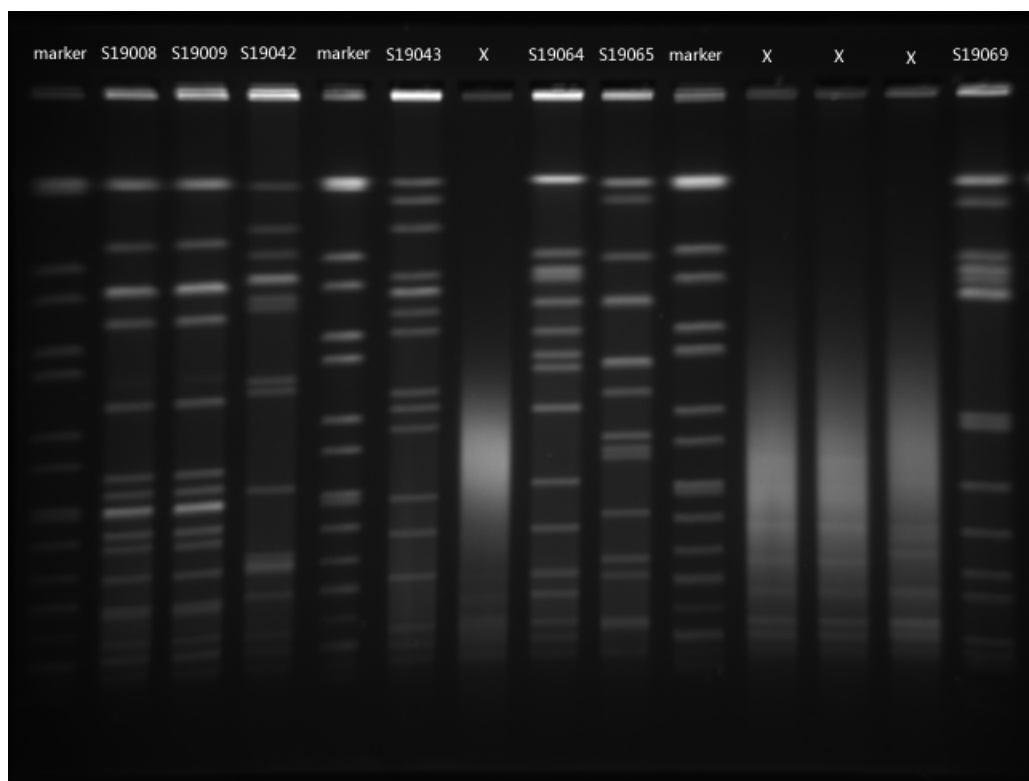

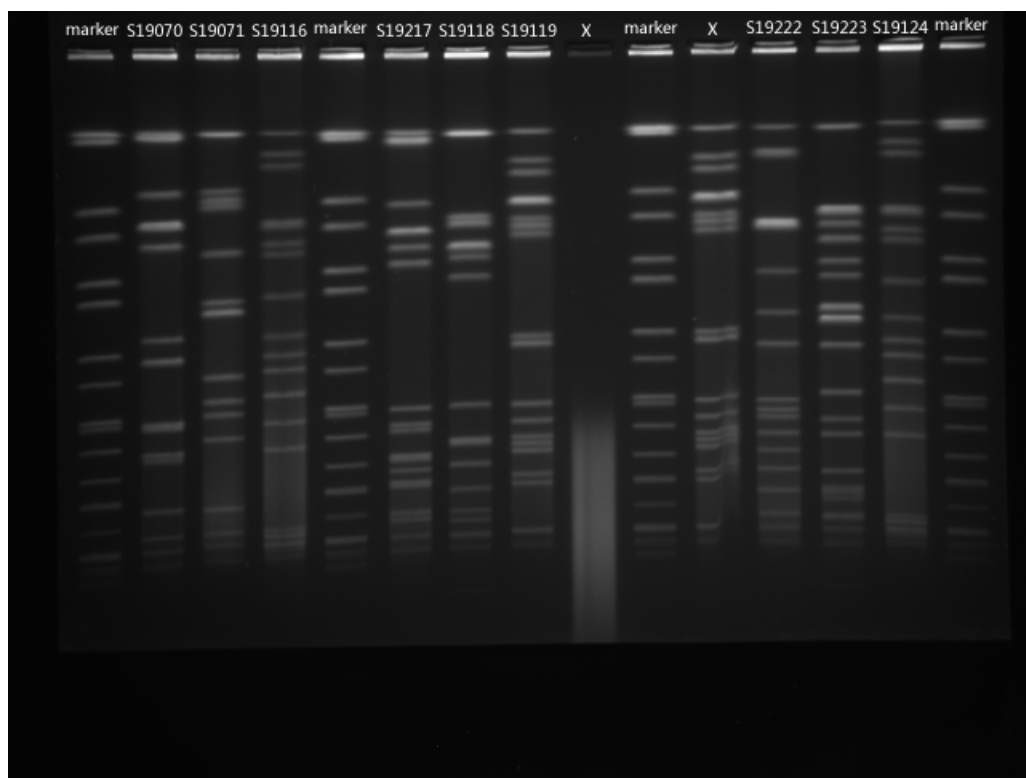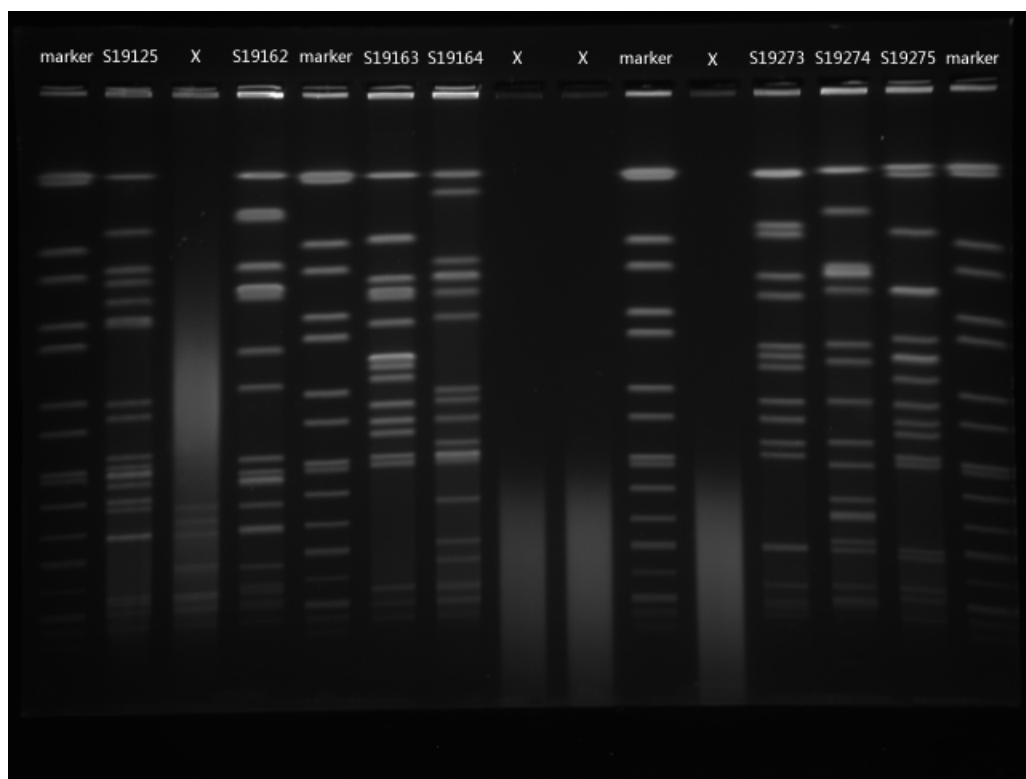

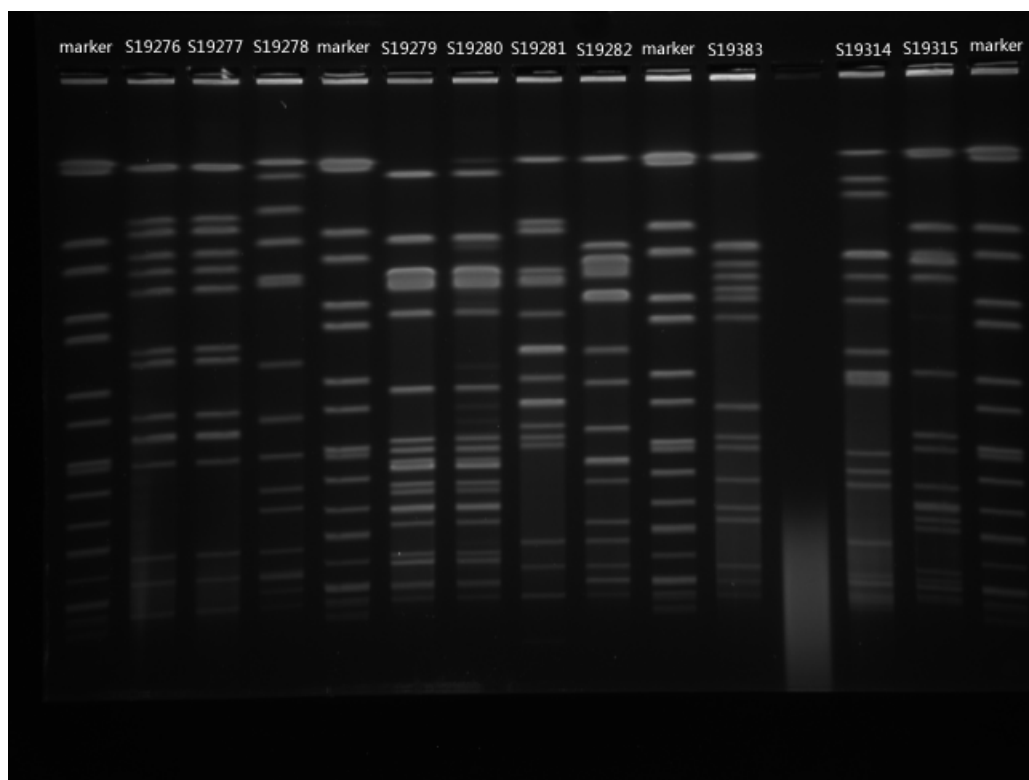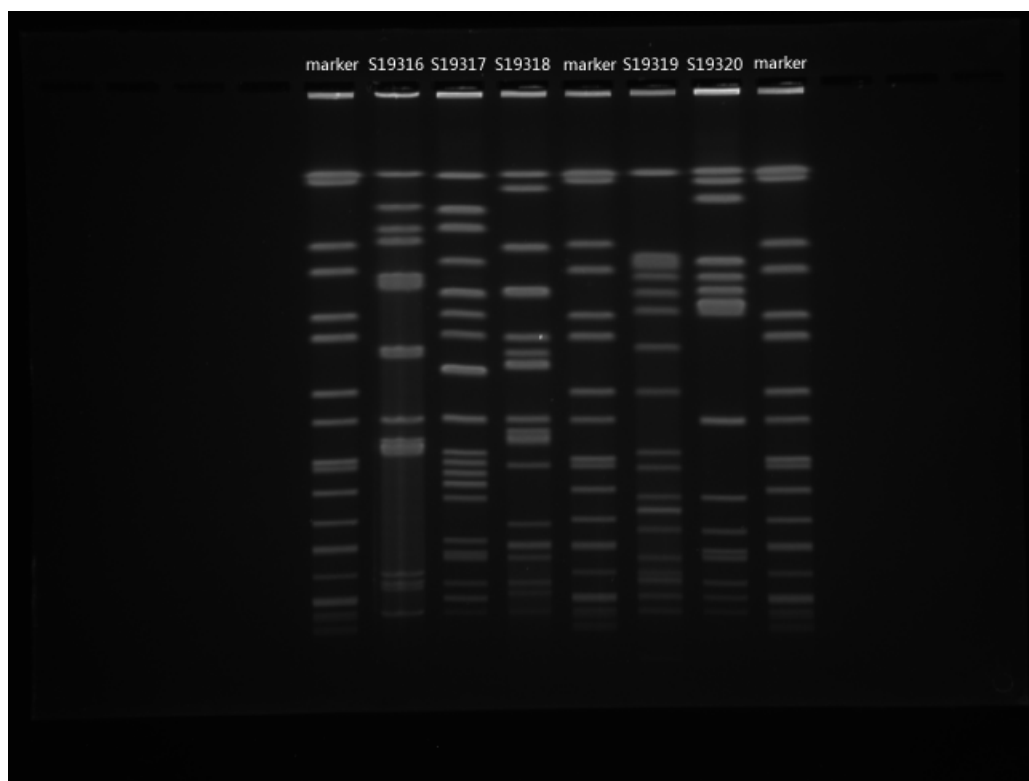

Supplement: S1 Raw images — (PDF) [file pone.0240143.s001.pdf]
